# Normal tissue toxicity after small field hypofractionated stereotactic body radiation

**DOI:** 10.1186/1748-717X-3-36

**Published:** 2008-10-31

**Authors:** Michael T Milano, Louis S Constine, Paul Okunieff

**Affiliations:** 1 Department of Radiation Oncology, University of Rochester Medical Center, Rochester, NY 14642, USA

## Abstract

Stereotactic body radiation (SBRT) is an emerging tool in radiation oncology in which the targeting accuracy is improved via the detection and processing of a three-dimensional coordinate system that is aligned to the target. With improved targeting accuracy, SBRT allows for the minimization of normal tissue volume exposed to high radiation dose as well as the escalation of fractional dose delivery. The goal of SBRT is to minimize toxicity while maximizing tumor control. This review will discuss the basic principles of SBRT, the radiobiology of hypofractionated radiation and the outcome from published clinical trials of SBRT, with a focus on late toxicity after SBRT. While clinical data has shown SBRT to be safe in most circumstances, more data is needed to refine the ideal dose-volume metrics.

## Introduction

Stereotactic body radiation therapy (SBRT) uses novel technologies to more accurately localize radiation targets. The word stereotaxis is derived from the Greek *stereos*, meaning solid (i.e. three-dimensional) and *taxis*, meaning order (i.e. arrangement or orientation); *stereotaxis *refers to movement in space. *Stereotactic*, combing the Greek *stereos *with the latin *tactic*, meaning "to touch," is the favored terminology. As the name implies, SBRT utilizes a three-dimensional coordinate system to achieve more accurate radiation delivery.[1,2] With SBRT, the radiation planning margins accounting for set-up uncertainty are minimized. This allows for greater dose-volume sparing of the surrounding normal tissues, which enables the delivery of higher fractional doses of radiation (hypofractionation). With SBRT, discrete tumors are treated with the primary goal of maximizing local control (akin to surgical resection) and minimizing toxicity. Arguably, SBRT has the potential to achieve better tumor control than a limited resection (i.e. resection without wide surgical margins) due to the penumbra dose around the target which targets microscopic extension of disease.[3]

SBRT has been defined as hypofractionated (1–5 fractions) extracranial stereotactic radiation delivery, [1,2,4,5] though arguably SBRT is more simply defined as a radiation planning and delivery technique in which a three-dimensional orientation system is used to improve targeting accuracy, regardless of dose fractionation. When selecting the fractional and total SBRT dose, several clinical considerations are important, including: (1) predicted risks of late normal tissue complications; (2) predicted tumor control; (3) financial costs and time expenditure for treatment planning and delivery.

The long-term impact of hypofractionated dose delivery to small volumes of normal tissues is not well understood, and certainly more clinical studies with longer follow-up are needed to better define the variables associated with risks of late toxicity.

### Technical aspects of SBRT

SBRT requires a means to detect and process a three-dimensional array. Various three-dimensional coordinate systems can be used, including internal fiducials, external markers and/or image guidance. Image guided radiation therapy (IGRT), with daily CT imaging, ultrasound and/or orthogonal x-rays can assist in targeting accuracy.

Several other tools can be used to improve immobilization including stereotactic body frames, abdominal compression devices and vacuum bags. Respiratory gating, which allows for the radiation beam to be turned off when respiratory movements place the target outside of the pre-determined positioning parameters, and for radiation to resume when the target falls back within the accepted alignment, can help improve targeting. Controlled respiration, such as relaxed breath-hold or shallow breathing can also reduce set-up uncertainty.[6] Some SBRT systems (such as Cyberknife^®^) track three-dimensional coordinates in real time, while the head of the accelerator realigns itself in real time to accommodate fluctuations in the target position.

The planning and delivery of SBRT generally uses multiple non-coplanar and/or arcing fields, directed at the radiation target. As result, the dose gradient is steeper than with conventional radiation, though the low dose region encompasses a larger volume and is irregularly shaped. The dose with SBRT is generally prescribed to the isocenter and/or isodose line encompassing the target, resulting in an inhomogeneous dose delivery in which the isocenter receives a greater dose than the periphery of the target. To reduce dose to surrounding tissues, a lower isocenter dose is selected and/or the dose is prescribed to a higher isodose line. With hypofractionated SBRT, versus conventional radiation, the absolute prescribed radiation dose is less (due to the use of larger, more biologically effective dose fractions); this lower absolute dose, in conjunction with the normal tissues being encompassed by lower isodose lines, provides a biologically sound rationale for using SBRT to reduce normal tissue exposure.[7]

### Radiobiology of hypofractionated radiation

The classic linear-quadratic model of cell survival after radiation is widely used to predict tumor response and normal tissue toxicity from fractionated radiation. Though the linear-quadratic model has limitations, including the over-estimation of cell killing from radiation,[8] it does provide insight into predicting tumor control and normal tissue toxicity, and is often used as the basis for determining fractionation schemes.[9] The validity of using the linear-quadratic model to predict late effects has been questioned, as it is a model derived from *in vitro *cell survival assays of cancer cell lines and is not necessarily expected to predict *in vivo *toxicity of normal tissues, in which alteration and/or injury of various cell types is of greater importance than cell survival.[10]

Generally, normal tissue effects are more greatly impacted by fraction size than are acute effects, which is why 1.8–2.0 Gy fractions are considered standard in the irradiation of most diseases in which the patient is expected to survive long enough to potentially experience late radiation-induced toxicity. Thus, with hypofractionated radiation, there is heightened concern about the risks of late toxicity, even when SBRT techniques are used to reduce the volume of normal tissue exposed to high doses.

It is generally accepted that unrepaired radiation-induced DNA damage results in mitotic death. However, at higher fractional radiation doses, other mechanisms may play a significant role as well. Interestingly, accounting for the overestimation of linear-quadratic model in predicting tumor control (i.e. poorer control than expected) with large fractional doses, and accounting for the hypoxic fraction of tumors, and the relative radiation resistance associated with hypoxia, hypofractionation actually results in a greater than expected tumor control, suggesting that novel mechanisms which can overcome hypoxia may play a role with hypofractionation.[11]

Researchers from Memorial Sloan Kettering have shown endothelial apoptosis becomes significant above a ~8–10 Gy single dose threshold (albeit fractionated regimens were not compared to single dose treatments).[12] Endothelial apoptosis results in microvascular disruption and death of the tissue supplied by that vasculature.[13] Radiation, and perhaps higher fractional doses of radiation, may also play a role in stimulating an immune response. Radiation-induced stem cell depletion is also likely important. Stem cells can migrate into the radio-ablated tissue from neighboring undamaged tissue.

SBRT is well suited for the sparing of tumors involving or abutting parallel functioning tissues (i.e. kidneys, lung parenchyma and liver parenchyma, in which functional subunits are contiguous, discrete entities).[1,4] SBRT reduces the organ volume, and thus the absolute number of parallel functioning subunits destroyed by radiation. Because of an organ reserve, with redundancy of function, the undamaged functional subunits can maintain the organ function (as occurs in lung, liver and kidney) and/or regenerate new functional subunits (as occurs in liver). Serial functioning tissues (i.e., spinal cord, esophagus, bronchi, hepatic ducts and bowel, which are linear or branching organs, in which functional subunits are undefined) may also benefit from reduced high-dose volume exposure, though there is heightened concern about radio-abalting these tissues because of the potential devastating, irreversible downstream effects that can occur from damage to upstream portions of the organ.[1,4] Stem cell migration may be of greater importance with serial functioning tissue because unrepaired radiation-induced damage cannot be compensated for by the function of the undamaged organ. Though small volumes of serially functioning tissues, such as spinal cord, can safely receive suprathreshold doses,[14,15] the volume and anatomical regions which can receive suprathreshold dose are not well characterized, nor is the impact of inhomogenous dose delivery.[16]

### Review of select clinical trials using hypofractionated SBRT

Extracranial SBRT has been used in the treatment of tumors involving many organs, including lungs, liver, pancreas, kidneys, adrenals, spine and other musculoskeletal tissues.[2,17-20] SBRT techniques have also been used to safely treat primary prostate cancer.[21,22] Most studies report acute toxicity of SBRT, though many also discuss late toxicity.

It is critical to understand the dose-volume metrics that are important in predicting late toxicity in normal tissues such as spinal cord, esophagus, stomach, bowel, liver, kidneys and lungs.[23] Unfortunately, with SBRT, late clinical outcome data is limited, and thus comprehensive evidenced-base dose-volume constraints are not available. With increasing clinical experience, these constraints are likely to become better formalized. The total dose, fractional dose, volume of normal tissue exposed to high doses of radiation, and location of the target are critical variables in predicting late toxicity. However, host and tumor variables, which are presently not well characterized, are also likely relevant. The remainder of this paper reviews the published clinical experience of SBRT. Papers focusing on normal tissue effects after SBRT, particularly late toxicity with longer follow-up (when available), were selected for this review.

#### Lung

SBRT in commonly used to treat lung tumors, including primary lung cancer as well as limited metastases, in patients who are medically inoperable or who refuse more invasive techniques. Radiation is arguably the safest option for tumors abutting large vessels and central structures. Table 1 (Additional File 1) summarizes the toxicity, prescribed dose and dose-volume constraints in selected studies described below.

Acute and mild fatigue, malaise, cough and dermatitis are common. Acute esophagitis can occur with SBRT of central tumors.[24] Acute radiographic pneumonitis commonly occurs, though grade ≥3 pneumonitis is rare. Late toxicity is relatively uncommon. Reported late grade ≥3 toxicity ranges from 0–7%. Examples of grade ≥2 late toxicity include pneumonitis, [25-28] chronic cough,[29,30] pulmonary bleeding/hemoptysis,[31,32] bronchial fistula,[33] pulmonary function decline,[25,32] pneumonia,[32] pleural effusion,[25-27,32] airway narrowing, stricture or obstruction,[30,34,35] tracheal necrosis,[36] chest wall pain and/or rib fracture. [25,26,30,33,37-42] brachial plexopathy,[42,43] and esophageal ulceration.[31,37]

#### Select studies

At the University of Rochester, 49 patients were treated with SBRT for limited metastases in the thorax.[44] With a mean follow-up of 18.7 months, toxicity (acute and late) was as follows: grade 1–2 (mostly self-limited cough) in 41%; grade 3 (non-malignant pleural effusion successfully managed with pleurocentesis and sclerosis) in 1 patient; and no grade 4–5 toxicity. Pulmonary toxicity did not correlate with the volume of lung receiving >10 Gy or 20 Gy (V20).

In a Phase I study from Indiana University, 47 patients with medically inoperable Stage I non-small cell lung cancer (NSCLC) were treated with 3 fractions of SBRT, with the fractional dose escalated in 2 Gy increments, starting with 8 Gy fractions.[36,45] The mean follow-up was 27 and 19 months for Stage IA and IB NSCLC. Six patients developed acute radiation pneumonitis requiring steroids. Three of 5 patients receiving 24 Gy fractions developed grade 3–4 pneumonitis (n = 2) or tracheal necrosis (n = 1), though the timing of these toxicities is not discussed.[36] Seventy patients with inoperable Stage I NSCLC enrolled on a subsequent Phase II study of 60–66 Gy in 3 fractions.[32] Eight patients developed grade 3–4 toxicity 1–25 months after SBRT; including pulmonary function decline, pneumonia, pleural effusion, apnea, and dermatitis. Six patients experienced grade 5 toxicity 0.6 – 20 months (median 12) after SBRT: 4 from pneumonia, 1 from pericardial effusion and another from massive hemoptysis. The extent to which SBRT contributed to the death in these patients cannot be determined. Central and hilar tumor location versus peripheral tumors (p = 0.004) and tumor size 10 ml (p = 0.017) were adverse predictors of grade 3–5 toxicity.

In a Phase I study from Stanford University, 32 patients with a solitary metastasis or Stage I NSCLC received single fraction SBRT, escalated from 15–30 Gy. Central tumor location, dose >15 Gy and tumor volume were associated with a greater risk of severe to fatal toxicity.[46] At a median follow-up of 18 months, 3 patients died 5–6 months after SBRT from radiation pneumonitis (n = 2) and tracheo-esophageal fistula (n = 1).

Based on the Indiana University experience, the Radiation Therapy Oncology Group treated 55 patients with peripheral Stage I NSCLC with 60 Gy in 20 Gy fractions. With median follow-up of 8.7 months, 7 patients developed grade 3 pulmonary/upper respiratory toxicity and 1 developed grade 4 toxicity.[47]

In a retrospective study from Technical University, Germany, 68 patients with Stage I NSCLC received 30–37.5 Gy in 10–12.5 Gy fractions for peripheral tumors or 35 Gy in 7 Gy fractions for central thoracic tumors.[26] Acute radiation pneumonitis occurred in 36% of patients, while only 1 patient developed late grade 3 radiation pneumonitis (at 4 months) which progressed to fibrosis. One patient developed a grade 2 soft tissue fibrosis. With a mean follow-up of 17 months, no other grade >2 toxicity was observed.

In a study from Hong Kong, 20 patients received 45–60 Gy in 3–4 fractions of 12–18 Gy for peripheral Stage I NSCLC.[40] No grade ≥2 acute or late toxicity was observed. Four patients received fractional doses >6 Gy to the esophagus. The maximal dose to the trachea and mainstem bronchus was 42.6 Gy in 14.2 Gy fractions (with ≤0.5 ml >12 Gy) in 1 patient; 2 others received >10 Gy per fraction and 4 others received >8 Gy per fraction. The maximal dose to the aorta was 59.1 Gy in 19.7 Gy fractions (with ≤3.3 ml >15 Gy) in 1 patient; 2 others received >10 Gy per fraction and 3 others received >8 Gy per fraction. The maximal dose to the heart was 40.4 Gy in 10 Gy fractions in 1 patient; 1 other received >10 Gy per fraction and 2 others received >8 Gy per fraction.

#### Radiation pneumonitis

Since the volume of lung exposed to clinically significant doses with SBRT is small, few pulmonary complications have yet to be observed by our group or others. As a result, it is difficult to ascertain dose-volume metrics to predict the risk of clinically significant radiation pneumonitis. Some studies have demonstrated the risk of radiation pneumonitis developing later (median of ~5 months) after SBRT versus after conventional radiotherapy.[27,28] A Japanese study has shown that a higher conformality index (less conformal plan) is significantly associated with a higher risk of pneumonitis, while other dose-volume metrics (i.e. mean lung dose and volume of lung exceeding incremental does) are not.[28] The V20 in that study ranged from 1–11%. In the study from the University of Rochester, in which pulmonary toxicity did not correlate with V20, the V20 ranged from 1–34%, with a median of 10%. Arguably the variance in V20 in these studies may not be large enough to conclude that V20 is not a significant predictor of radiation pneumonitis, since a V20 in the 30–40% range with standard fractionation is associated with increased risk of symptomatic pneumonits.[23] The standard dose-volume metrics used to predict radiation pneumonitis, such as V20, V13 and mean lung dose, may still be relevant.

#### Pulmonary function

For the most part, SBRT does not significantly impact pulmonary function, and in some patients pulmonary function may improve after SBRT.[37,48] Pulmonary function decline may be asymptomatic or transient in some patients.[45,49] In a study from Aarhus University, late dyspnea was not correlated to any dose-volume parameters, and no consistent temporal variations of dyspnea after SBRT were observed.[50] Worsening dyspnea was more attributable to pre-existing chronic obstructive pulmonary disease as opposed to late radiation effects. In a study of 70 patients from Indiana University, neither poor baseline values of forced expiratory volume in 1 second (FEV1) nor diffusing capacity of the lung for carbon monoxide (DLCO) predicted for time to first Grade ≥2 pulmonary toxicity or survival after SBRT.[51] While FEV1 did not significantly change over time, the DLCO significantly decreased by 1.11 ml/min/mm Hg/y. In a study from William Beaumont Hospital, FEV1 reductions occurred primarily at ~6 weeks, and remained stable thereafter, with a ~6–7% decline.[52] DLCO reductions occurred at >6 months. At 1-year, the DLCO was reduced ~16–21%, and mostly asymptomatic. The decrease in DLCO correlated with mean lung dose and V10–20, and was stable when corrected for alveolar volume, suggesting alveolar damage as a mechanism for change. There is no consensus on a safe lower limit of pulmonary function for SBRT. In the study from Indiana University, the pretreatment FEV1 ranged from 0.29–2.12 and the DLCO ranged from 3.5–23.05. Certainly, clinical judgment is needed to determine the safety of SBRT in any given patient, taking into account the pulmonary function, as well as the location and number of lesions.

#### Rib fracture/chest wall pain

Rib fractures can be asymptomatic, and therefore perhaps under-reported. In a study from Hong Kong, the dose to the chest wall in 3 patients who experienced asymptomatic rib fractures was 20–21 Gy in 3–4 fractions.[40] In a multi-institutional study, the risk of rib fracture from SBRT to peripheral lung lesions, ≤1.5 cm from chest wall, was a function of the absolute volume of chest wall receiving >30 Gy in 3–5 fractions.[41] No rib fractures occurred with <35 ml of chest wall receiving >30 Gy; at >35 ml, half of the patients developed rib fracture. Princess Margaret Hospital reported a 48% 2-year risk or rib fracture, mostly asymptomatic or mildly symptomatic, a median of 17 months after delivery of 54–60 Gy in 18–20 Gy fractions for tumors close (0–1.8 cm, median 0.4 cm) to the chest wall.[38] The median dose at the fracture site was 29–78 Gy (median 49). In a prospective Japanese study, 1 of 45 patients developed grade 2 chest wall pain after receiving a prescribed dose of 60 Gy in 7.5 Gy fractions to a peripheral tumor; the chest wall received a maximal dose of 48 Gy.[37]

#### Esophageal toxicity

With standard fractionation, the volume, length and surface of esophagus exposed to suprathreshold radiation increases the risk of toxicity.[23] SBRT can reduce the amount of esophagus exposed to therapeutic doses, though hypofractionated radiation delivery does raise concern for esophageal toxicity. Generally, the dose constraints adhered to for esophagus have proven to be safe (see Table 1 (Additional File 1)). In a prospective Japanese study, 1 of 45 patients developed grade 5 esophageal ulceration 5 months after receiving a prescribed dose of 48 Gy in 6 Gy fractions; in this patient, the esophageal maximum was 50.5 Gy and 1 cc of esophagus received >42.5 Gy.[37]

#### Brachial plexopathy

In an Indiana University study of 37 lesions in 36 patients with apical lung tumors treated to median dose of 57 Gy, the 2-year risk of brachial plexopathy was 46% after the brachial plexus received a biologically effective dose maximum of >100 Gy versus 8% for <100 Gy (*p *= 0.04).[43] Anther study reported brachial plexopathy in 1 of 60 patients due to significant volume of brachial plexus receiving 40 Gy in 4 fractions.[42]

#### Radiographic changes

Following SBRT, the lung parenchyma undergoes acute (occurring after weeks to months) and late (after 6 months) changes, reflected by characteristic radiographic findings,[27,53-55] and perhaps correlated to V7–10 and mean lung dose. [56] Acute radiation pneumonitis appears radiographically as diffuse or patchy consolidation and/or ground glass opacities. Late radiographic fibrosis can be linear and streaking or mass-like. The fibrosis can change in shape and extent; it can shrink and migrate centrally towards the hilum over the course of several months of follow-up imaging.[27,55] It can also grow, appear as abnormal opacities, and/or potentially mimic recurrent tumors.[27,57,58] While late radiographic changes reflect fibrosis, the clinical significance of these changes is not known. Radiographic bronchial/tracheal wall thickening (with or without clinical airflow restriction) can also be seen.[34]

In a study from Hiroshima University, patients were followed with serial CT scans after receiving 48–60 Gy in 3.85–12 Gy fractions. Patients who developed grade >2 radiation pneumonitis, were more likely to have had acute diffuse consolidation or no evidence of acute radiographic changes (versus patchy consolidation or ground glass opacity changes).[54] The late changes, classified as modified conventional pattern (consolidation, volume loss and bronchiectasis), mass-like pattern (focal consolidation around tumor site) and scar-like pattern (linear opacities and volume loss), developed in 62%, 17% and 21% respectively. Among those lesions developing acute diffuse consolidation, 80% proceeded to develop to a modified conventional pattern of late changes; among those lesions with no acute densities, 59% developed a scar-like pattern of late changes.

In a study from Kyoto University, late changes (after a dose of 48 Gy in 12 Gy fractions) developed as patchy consolidation (within irradiated lung, not conforming to SBRT field) in 8%, discrete consolidation (within SBRT field, not outlining shape of field) in 27% and solid consolidation (outlining SBRT field) in 65%.[53] The shape of the radiation changes were described as wedge (35%), round (35%) and irregular (29%); the extent of fibrotic change was described as peripheral (48%), central (6%), both (39%) and skip lesion(s) isolated from the tumor (6%).

#### Liver

SBRT in commonly used to treat liver tumors, including hepatocellular carcinoma as well as limited metastases, in patients who are medically inoperable, who refuse more invasive techniques, whose disease is unresectable and/or who have several lesions. Table 2 (Additional file 1) summarizes the toxicity, prescribed dose and dose-volume constraints used in selected studies described below.

Acute mild fatigue, malaise, nausea, diarrhea and dermatitis are common. Grade ≥3 toxicity, including hepatic failure, bowel perforation or obstruction and gastrointestinal bleeding, is rare. In the rare situations of hepatic failure, it is often difficult to determine whether hepatic failure resulted from radiation or tumor progression.

#### Select studies

At the University of Rochester, 69 patients were treated with SBRT for limited metastases of the liver. At a median follow-up of 14.5 months, grade 1–2 elevation of liver function tests occurred in 28% of patients, and no grade ≥3 toxicity was observed.[59] Clinically insignificant radiographic changes were seen in all patients.

In a collaborative Phase I study, the University of Colorado and Indiana University enrolled 18 patients with 1–3 liver metastases treated with three fractions of SBRT.[60] No patients developed grade >2 toxicity. Late radiographic changes of well circumscribed hypodense lesions were commonly seen, corresponding to the 30 Gy dose distribution. In a follow-up analysis, including an additional 18 patients treated on a Phase II study of 3 fractions of 20 Gy, 1 patient developed subcutaneous tissue breakdown; no radiation-related liver toxicity occurred.[61]

In a study from Aarhus University, 44 patients with liver metastases from colorectal cancer received a dose of 45 Gy in 15 Gy fractions. Acute toxicity (<6 months after SBRT) included grade 3 colonic ulceration (n = 1), grade 3 duodenal ulceration (n = 2), grade 3 skin ulceration (n = 2), grade 3–4 pain (n = 11), grade 3 nausea (n = 2) and grade 3 diarrhea (n = 2). One patient died from hepatic failure <2 months after SBRT. Late toxicity was not explicitly discussed.[62] Grade 3 gastric and duodenal mucosal ulceration 3 months after SBRT was also reported in 2 of 48 patients in a recent Italian study, in which patients received 30–36 Gy in 3 fractions.[63]

Princess Margaret Hospital treated 41 patients with primary hepatocellular or intrahepatic biliary cancer on a Phase I study of 24–60 Gy in 6 fractions.[64] Using normal tissue complication modeling, patients were stratified into 3 different dose escalation groups, based on the effective liver volume to be irradiated. Acute (<3 months) elevation of liver enzymes occurred in 24% of patients, acute grade 3 nausea occurred in 7% and acute transient biliary obstruction occurred in 5% patients. There was one late death from gastrointestinal bleeding of a duodenal-tumor fistula and one patient required surgery for a bowel obstruction; both late toxicities were exacerbated by (and perhaps attributable to) recurrent disease.

#### Pancreas

Locally advanced pancreatic cancer has a grave prognosis, with a high likelihood of metastatic and local progression. Radiation can palliate or prophylactically palliate symptoms from local progression, such as biliary obstruction, bowel obstruction and splanchnic nerve pain. SBRT may afford an advantage in terms of improved local control, reduced volume of normal tissue exposure and shorter treatment duration.

Table 3 (Additional file 1) summarizes the toxicity, prescribed dose and dose-volume constraints used in the studies described below.

#### Select studies

Aarhus University conducted a Phase II study in which 22 patients with unresectable pancreatic cancer received 45 Gy in 15 Gy fractions.[65]. All evaluable patients developed acute (14 days post- treatment) decline in performance status and nausea, and most developed acute to subacute pain. Other grade 2–4 toxicities included diarrhea, and gastrointestinal mucositis, ulceration and perforation. Whether toxicity was related to SBRT or disease progression could not be assessed. Poor survival precluded a late toxicity analysis.

Stanford University conducted a Phase I in which 15 patients with unresectable pancreatic cancer received single fraction SBRT, escalated from 15 to 25 Gy.[66] No acute grade ≥3 toxicity was observed; late toxicity and symptom control were not explicitly reported, presumably due to limited follow-up (median 5 months) and poor survival (median 11 months). In a subsequent Phase II study, 16 patients received 45 Gy with intensity modulated radiotherapy followed by a single 25 Gy SBRT fraction.[67] Acute grade 3 toxicity included gastroparesis in 2 patients (one prior to receiving SBRT). Late toxicity occurred in some patients (number not explicitly reported) who developed grade 2 duodenal ulceration 4–6 months after SBRT. In a later report, the authors document late gastrointestinal bleeding (unknown cause) and duodenal obstruction occurring in the same patient.[68]

The reported tolerability of SBRT by Stanford University conflicts with the excessive toxicity reported by Aarhus University. Perhaps these differences are attributable to different dose fractionation, different treatment design (i.e. Stanford University uses respiratory tracking), differences in patient population (i.e. tumor volumes were appreciably larger in Aarhus University study) and/or differences in failure pattern.

#### Radiation induced histo-pathologic changes

In a study from Stanford University, the pathologic changes after SBRT to the pancreas were characterized in 4 patients who underwent an autopsy 5–7 months after SBRT.[68] The primary tumors developed extensive fibrosis, tumor necrosis, ischemic necrosis widespread vascular injury (fibrinous exudate of arterial wall, necrosis and luminal occlusion) and sparse residual cancer cells. Stromal changes included fibrosis, atypical fibroblasts and fibrin deposition. Lymph nodes within the SBRT field were depleted of lymphocytes. In 1 patient, the adjoining colorectal mucosa, estimated to have received 4–11.5 Gy, developed a mucosal exudate with possible pseudomembrane formation and submucosal vascular damage.

#### Spine

Spinal metastases are quite common and are readily palliated with radiation. The commonly prescribed doses of 20 – 40 Gy in 2.5 – 4 Gy fractions effectively palliates spinal metastases, with safe dose exposure to the spinal cord. The prescribed dose of 20 – 40 Gy with these larger fraction sizes is generally accepted to be at the spinal cord tolerance (though certainly below the TD 5/5).[23] Additional radiation can be delivered to maximize tumor control or to treat recurrent disease, albeit with greater risks of spinal cord toxicity.[69] In patients with previously irradiated, symptomatic spinal metastases, SBRT is well suited to deliver additional radiation to the vertebral body while minimizing spinal cord dose. While hypofractionation in this situation is counter-intuitive, early clinical data has shown it to be tolerable, albeit with limited patient follow-up.

Several studies have demonstrated excellent palliation using single fraction (spinal radiosurgery) [70-77] and hypofractionated SBRT [75,78-80] to treat spinal metastases, using tools such as intensity modulated radiation,[72,73,77-79,81] and IGRT, [82] to minimize spinal cord dose. At least one report has suggested that acute toxicity using SBRT is perhaps better than conventional radiation.[83] Late toxicity is difficult to assess in this population of patients due to the poor survival of patients with metastatic disease. However, it appears that myelopathy and radiculopathy rarely occur.[84] Most institutions try to achieve a spinal cord maximum dose <10 Gy.[83] A recent multi-institutional pooled analysis has shown that radiation myelopathy has only been documented to occur after exceeding a fractional dose maximum of 10 Gy to the spinal cord and/or a biologically effective dose of 60 Gy in 2 Gy fractions; other dose-volume paramaters such as dose to 1–5 ml of spinal cord were not significant in predicting radiation myelopathy.[85] More rigid dose constraints have yet to be published. A recent paper offers a comprehensive review of spinal radiosurgery. [77] Select studies are discussed below, with a focus on treatment related toxicity.

#### Select studies

Henry Ford Hospital published the planning constraints and outcome of single fraction SBRT in the treatment of 233 lesions in 177 patients. Their data suggests that a dose constraint of 10 Gy to <10% of the contoured spinal cord (6 mm above and below the target) is safe, and that small volumes (<1% of the contoured cord) can safely receive higher maximal doses, perhaps up to 20 Gy.[70,71] One of 177 patients developed radiation related spinal cord injury, resulting in mild unilateral lower extremity weakness (4 out of 5 strength) that responded to steroids.

In a study from Memorial Sloan Kettering, 103 lesions in 93 patients were treated with single fraction SBRT; the prescribed dose was 18–24 Gy to the PTV, with the spinal cord limited to 12–14 Gy. [86] Late toxicity included radiographic evidence of vertebral body fracture in the absence of tumor in 2 patients and tracheoesophageal fistula requiring surgery in 1 patient.

The University of Pittsburgh recently updated their experience of single dose SBRT in 393 patients with 500 lesions. The prescribed dose was 12.5–20 Gy around the periphery of the targeted lesions, allowing for only a small volume of spinal cord to exceed 8 Gy. No acute or late neurotoxicity was observed, and no late toxicity was reported after a follow-up of 3–53 (median 21) months.

### Recommendations

Deriving standard acceptable maximally effective and minimally toxic dose fractionation schemes presents a challenge, even with the available published outcome data. In part, this complexity arises from not only the different dose-fractionation schemes used, but also in differences in how the dose is prescribed. For example, a fractional dose of 20 Gy delivered to the isocenter is appreciably less than a fractional dose of 20 Gy delivered to the 80% idosdose line and/or periphery of the PTV. Tables 1–3 (Additional file 1) summarize how the dose was prescribed in many of the studies discussed above. These tables also summarize the late toxicity (as well as acute toxicity if the timing of the toxicities was not elaborated). While some studies provided a correlation of toxicity with dose-volume parameters of the affected normal tissue, most did not. Acknowledging these limitations, Tables 4–5 (Additional file 1) attempt to offer recommendations for safe SBRT hypofractionated dose exposure to small volumes of normal tissues. It should be appreciated that these are general guidelines derived from the literature as discussed above. For the most part, the volume of normal tissue exceeding these tolerance doses is not well described, but certainly every effort should be made to minimize the volume exposed to therapeutic or close to therapeutic dose. Tables 1–2 (Additional file 1) do offer the dose-volume constraints used in published studies and the recent RTOG 0236 and ongoing RTOG 0438 studies

## Conclusion

SBRT reduces the volume of normal tissue exposed to therapeutic doses, allowing for larger fractional dose delivery. Recent clinical data has demonstrated the efficacy and safety of SBRT in the treatment of tumors in several body sites. Further study and longer follow-up are needed to ascertain the dose-fractionation schedule that optimizes tumor control while minimizing toxicity, and to better understand the optimal normal tissue dose-volume constraints. CURED, a recently formed multi-institutional, international collaborative group stemming from the Late Effects of Normal Tissue (LENT) conferences, is actively investigating late effects after cancer therapy, and is potentially well-equipped to further investigate late toxicity after SBRT.

## Competing interests

The authors declare that they have no competing interests.

## Authors' contributions

All authors contributed to drafting the manuscript and all authors reviewed and approved the final manuscript.

## Author information

MM is an Assistant Professor in the Department of Radiation Oncology at the University of Rochester, whose clinical and research interests include the treatment of limited metastases with stereotactic body radiation.

PO is Professor and Chairman of the Department of Radiation Oncology at the University of Rochester. In addition to basic science research investigating the amelioration of radiation-related toxicity, he has an interest in the study and treatment of patients with limited metastases.

LSC is Professor and Vice-chairman of the Department of Radiation Oncology at the University of Rochester. He has a long-standing interest in the study of cancer survivorship and treatment related late effects. Both LSC and PO are involved in developing an international, multi-institutional cooperative group, CURED, devoted to cancer survivorship and late effects.

## Supplementary Material

Additional file 1 Toxicity and dose-volume constraints in select studies of patients undergoing stereotactic body radiotherapy for thoracic lesions Click here for file
