# Supplementary material for: Normal tissue toxicity after small field hypofractionated stereotactic body radiation
Source: Radiat Oncol. 2008 Oct 31;3:36. doi: 10.1186/1748-717X-3-36 (PMC2596155; doi:10.1186/1748-717X-3-36)
Supplement: Additional file 1 — Toxicity and dose-volume constraints in select studies of patients undergoing stereotactic body radiotherapy for thoracic lesions [file 1748-717X-3-36-S1.doc]

**Table 1:**

Toxicity and dose-volume constraints in select studies of patients undergoing stereotactic body radiotherapy for thoracic lesions

| **Institution** | **patient population** | **prescribed dose** | **Dose volume constraints** | **Grade ≥3 late toxicity** |
| --- | --- | --- | --- | --- |
| U. Rochester ^19,20,44^ | 49 patients with limited metastases | • 50 Gy / 10 fractions  in most patients  • Rx to isocenter  • PTV = GTV + 7-10 mm  • PTV covered by 80% IDL | • ≥1000 ml of tumor-free lung  • 60% of lung ≤2.0 Gy / fraction (patients with healthy lungs)  • 70% of the lung or 800 ml ≤1.7 Gy / fraction  (patients with chronic obstructive lung disease)  • spinal cord: dose to center ≤2 Gy / fraction and ≤45 Gy total  dose to surface ≤54 Gy. | 1 patient with a late grade 3 pleural effusion. |
| Indiana U. ^36,45^ | 47 patients with Stage I NSCLC | • 24 → 72 Gy / 3 fractions  • PTV = GTV + 5-10 mm  • Rx to 80% IDL  • 95% of PTV covered by 80% IDL | • spinal cord maximum: ≤6 Gy / fraction | 7 patients with grade 3-4 toxicity, including pneumonitis (n=3), pericardial effusion, tracheal necrosis, hypoxia and dermatitis.  Timing of toxicity not reported |
| Indiana U. ^32^ | 70 patients with Stage I NSCLC | • 60 – 66 Gy / 3 fractions  • Rx as above | • as above | 14 patients with Grade 3-5 toxicity, 0.6-25 months after SBRT  Grade 3-4 toxicity in 8 patients: pulmonary function decline, pneumonia, pleural effusion, apnea, and dermatitis.  Grade 5 toxicity in 6 patients:  pneumonia (n=4), pericardial effusion, massive hemoptysis. |
| Technical U. ^26^ | 68 patients with Stage I NSCLC | • 30-37.5 Gy / 3 fractions (peripheral)  • 35 Gy / 5 fractions (central)  • Rx to 60% IDL  • PTV covered by 60% IDL  • PTV derived from tumor motion | • spinal cord maximum: ≤15 Gy / 3 fractions or 20 Gy / 5 fractions  • esophagus maximum: ≤21 Gy / 3 fractions or 27.5 Gy / 5 fractions | 1 patient with late pneumonitis and pulmonary fibrosis.  1 patient with grade 3 soft tissue fibrosis. |
| Hokkaido U. ^37^ | 45 patients with Stage I NSCLC and limited metastases | • 48-60 Gy / 8 fractions  • Rx to isocenter  • 80% IDL covers PTV  • PTV = ITV + 1 cm | • spinal cord maximum: ≤3.75 Gy / fraction  • esophagus maximum: ≤5 Gy / fraction  • brachial plexus maximum: ≤4.625 Gy / fraction | 1 patients with grade 3 esophageal ulceration |
| Tuen Mun Hospital ^40^ | 20 patients with Stage I NSCLC | • 45-60 Gy / 3-4 fractions  • PTV = GTV + 7-10 mm  • Rx to 85-90% IDL  • 95% of GTV covered by Rx IDL | • spinal cord maximum: ≤6 Gy / fraction  • esophagus maximum: ≤8 Gy / fraction  • retrospectively analyzed (see text) | no grade ≥3 toxicity observed |
| Stanford U. ^46^ | 32 patients with Stage I NSCLC and solitary metastasis | • 15-30 Gy / 1 fraction  • PTV = GTV + 2-5 mm  • 95% of PTV covered by Rx IDL | • spinal cord maximum: ≤8 Gy  • 2/3 total lung to receive ≤5 Gy  • 1/2 heart to receive ≤10 Gy  • esophageal maximum ≤20 Gy  • 1/2 esophageal volume to receive ≤10 Gy  • brachial plexus maximum: ≤10  • 1/2 liver volume to receive ≤7.5 Gy | 1 patient with Grade 3 pneumonitis  3 patients with grade 5 toxicity, including pneumonitis (n=2) and tracheal-esophageal fistula (n=1). |
| RTOG 0236 ^47^ | 55 patients with Stage I NSCLC | • 60 Gy / 3 fractions  • PTV = GTV + 7-10 mm  • 95% of GTV covered by Rx IDL | • spinal cord maximum: ≤6 Gy / fraction  • esophagus maximum: ≤9 Gy / fraction  • brachial plexus maximum: ≤8 Gy / fraction  • heart maximum: ≤10 Gy / fraction  • trachea and bronchus maximum: ≤10 Gy / fraction | 8 patients with acute and late grade 3-4 pulmonary/upper respiratory toxicity |

IDL = isodose line

Rx = prescribed

GTV = gross tumor volume

ITV = internal target volume

PTV = planning target volume

NSCLC = non small cell lung cancer

**Table 2:**

Toxicity and dose-volume constraints in select studies of patients undergoing stereotactic body radiotherapy for liver lesions

| **Institution** | **patient population** | **prescribed dose** | **Dose volume constraints** | **Grade ≥3 toxicity** |
| --- | --- | --- | --- | --- |
| U. Rochester ^19,20,59^ | 69 patients with limited metastases | • 50 Gy / 10 fractions  in most patients  • Rx to isocenter  • PTV = GTV + 7-10 mm  • PTV covered by 80% IDL | • ≥1000 ml of tumor-free liver  • 60% of liver ≤30 Gy (patients with healthy livers)  • 70% of liver ≤30 Gy (patients with macronodular sclerosis or hepatitis)  • ≤50% of the kidneys >16 Gy (with 2 functioning kidneys)  • ≤50% of kidney, >10% of total dose, at fraction size of <1.5 Gy  (with 1 functioning kidney).  • small bowel maximum ≤50 Gy.  • spinal cord: see Table 1 | no grade ≥3 toxicity |
| U. Colorado and  Indiana U. ^60^ | 18 patients with limited metastases | • 36 → 60 Gy / 3 fractions  • PTV = GTV + 5-10 mm  • Rx to 80%-90 IDL | • 700 ml of normal liver ≤15 Gy  • 67% of right kidney ≤5 Gy / fraction  • 35% of right and left kidney ≤5 Gy / fraction  • spinal cord maximum: ≤6 Gy / fraction  • stomach or small intestines maximum: ≤10 Gy / fraction | no grade ≥3 toxicity |
| U. Colorado and  Indiana U. ^61^ | 36 patients with limited metastases | • 60 Gy / 3 fractions  • Rx as above | • as above | 1 patient with grade 3 soft tissue necrosis and fibrosis. |
| Aarhus U. ^62^ | 44 patients with limited metastases | • 45 Gy / 3 fractions  • PTV = CTV + 10 mm  • 95% IDL encompass CTV  • 67% IDL encompass PTV | • ≤30% of the liver >10 Gy  • spinal cord maximum: ≤6 Gy / fraction  • dose to kidneys, intestines and stomach as low as possible. | Late toxicity not addressed |
| U. Firenze ^63^ | 41 patients with HCC or limited metastases | • 30-36 Gy / 3 fractions  • Rx to 90% IDL | • 700 cc of healthy liver <15 Gy | 2 patients with gastro-intestinal ulceration |
| Princess Margaret Hospital ^64^ | 41 patients with HCC or intrahepatic biliary ca. | • 24 → 60 Gy / 6 fractions  • PTV_primary_ = GTV + ≥5 mm  • PTV_secondary_ = CTV + 8 mm | • mean liver dose <22 Gy  • mean kidney dose <12 Gy  • maximum of 27 Gy to <0.5 ml of spinal cord  • maximum of 30 Gy to <0.5 ml of stomach and large bowel  • maximum of 40 Gy to <0.5 ml of heart | 1 patient with late bowel obstruction  1 patient death from bleeding duodenal-tumor fistula. |
| RTOG 0438 | limited metastases | • 35 → 50 Gy / 10 fractions  • PTV = GTV + 9 - 35 mm  • Rx to IDL covering PTV | • ≥1000 ml of tumor-free liver  • 70% of liver <27 Gy and 50% of liver <24 Gy  • <10% of kidney(s), ≥10 Gy  (with 1 functioning kidney or creatinine >2 mg/dl)  • <33% of kidney(s), ≥18 Gy  (with 2 functioning kidney and creatinine ≤2 mg/dl)  • spinal cord maximum: 34 Gy  • small bowel and stomach: ≤37 Gy to ≤1 cc volume | Results pending |

IDL = isodose line

Rx = prescribed

GTV = gross tumor volume

PTV = planning target volume

HCC = hepatocellular cancer

**Table 3:**

Toxicity and dose-volume constraints in select studies of patients undergoing stereotactic body radiotherapy for pancreatic cancer

| **Institution** | **patient population** | **prescribed dose** | **Dose volume constraints** | **Grade ≥3 toxicity** |
| --- | --- | --- | --- | --- |
| Aarhus U. ^65^ | 22 patients with UPC | • 45 Gy / 3 fractions  • PTV = CTV + 10 mm  • 67% IDL covers PTV | Not discussed | Nearly all patients with acute/subacute grade 2-4 nausea and pain  Grade 3-4 toxicities include diarrhea, nausea, pain, mucositis, ulceration. |
| Stanford U. ^66^ | 15 patients with UPC | • implanted fiducials  • 15-25 Gy / 1 fraction  • Rx IDL covers GTV | • 50% IDL covers only duodenal wall closest to the tumor  • mean dose to 5% of duodenum ≤22.5 Gy;  • mean dose to 50% of duodenum ≤14.5 Gy | No acute grade ≥3 toxicity |
| Stanford U. ^67^ | 16 patients with UPC | • 45 Gy- IMRT followed by  • 25 Gy / 1 fraction | For IMRT:  • 70% of the liver <15 Gy  • 70% of each kidney <15 Gy  • 95% of bowel <45 Gy  • spinal cord maximum <30 Gy | 2 patients with acute grade 3 gastroparesis  1 patient with late gastrointestinal bleeding and duodenal obstruction |

IDL = isodose line

Rx = prescribed

GTV = gross tumor volume

PTV = planning target volume

UPC = unresectable pancreatic cancer cancer

IMRT = intensity modulated radiation therapy

**Table 4:**

Recommendations for safe hypofractionated SBRT fractional doses to small volumes of serially arranged normal tissues

Number of fractions

Normal Tissue 1 3 5 8 10

Spinal cord 8-10 Gy 5-6 Gy 4-5 Gy 3-4 Gy 3 Gy

Trachea and Bronchi NR NR 7-9 6-7 4-5

Brachial plexus NR NR 8-10 6-7 5-6

Esophagus NR NR 6-8 4-5 3-4

Chest wall/ribs NR 10-15 6-8 6-7 5-6

Small bowel 10-12 10-12 6-8 5-6 4-5

* fractional dose

NR = not recommended to receive therapeutic or close to therapeutic doses with this number of fractions.

**Table 5a:** Recommendations for safe hypofractionated SBRT fractional doses to parallel arranged normal tissues

Number of fractions

Normal Tissue 1 3 5 8 10

Lung 20 Gy 20 Gy 8-10 Gy 7-8 Gy 5-7 Gy

Liver 25 20 8-10 7-8 5-6

These doses are expected to be safe with respect to risk of radiation necrosis

**Table 5b:** Recommendations for safe hypofractionated SBRT dose-volume metrics for parallel arranged normal tissues

Lung • 700 – 1000 ml of lung not involved with gross disease

• V20 of 25-30%

Liver • 700 – 1000 ml of liver not involved with gross disease

• two-thirds of normal liver <30 Gy

Kidney • minimize dose receiving >20 Gy

• two-thirds of one kidney <15 Gy (with another functional kidney)
